# Growth of Anisotropic Gold Nanoparticle Assemblies via Liposome Fusion

**DOI:** 10.3390/ma10111317

**Published:** 2017-11-17

**Authors:** Kouta Sugikawa, Tatsuya Kadota, Kotaro Matsuo, Kazuma Yasuhara, Atsushi Ikeda

**Affiliations:** 1 Graduate School of Engineering, Hiroshima University, Higashi-Hiroshima 739-8527, Japan; m150062@hiroshima-u.ac.jp (T.K.); m171813@hiroshima-u.ac.jp (K.M.); 2 Graduate School of Materials Science, Nara Institute of Science and Technology, Nara 630-0192, Japan; yasuhara@ms.naist.jp

**Keywords:** gold nanoparticles, anisotropic assembly, lipid membrane, liposomes

## Abstract

Anisotropic assembly of nanoparticles (NPs) has attracted extensive attention because of the potential applications in materials science, biology, and medicine. However, assembly control (e.g., the number of assembled NPs) has not been adequately studied. Here, the growth of anisotropic gold NP assemblies on a liposome surface is reported. Citrate-coated gold NPs adsorbed on liposome surfaces were assembled in one dimension at temperatures above the phase transition temperature of the lipid bilayer. Growth of the anisotropic assemblies depended on the heating time. Absorption spectroscopy and transmission electron microscopy revealed that the gradual growth was attributed to liposome fusion, which was strongly affected by the size of the gold NPs. This method enabled us to precisely control the number of NPs in each anisotropic assembly. These results will enable the fabrication of functional materials based on NP assemblies and enable investigations of cell functions and disease causality.

## 1. Introduction

When light interacts with a metal nanoparticle (NP), a unique surface plasmon resonance (SPR) frequency is observed [1]. The SPR strongly depends on the NP size and shape [1,2,3,4], its composition [3], and the nature of the dialectic materials surrounding the NPs [5,6,7]. Assembling NPs into desired dimensions or shapes is important for material science, bioscience, and medical applications [8,9,10,11,12,13].

Two- and three-dimensional NP assemblies have been made by solvent evaporation, Lagmuir–Blodgett transfer, self-assembled monolayers and multilayers, and layer-by-layer assembly. There are several excellent reviews [8,9,10,11]. However, anisotropic one-dimensional NP assemblies have been seldom reported because of preparation difficulties that arise from the isotropic structure and morphology of zero-dimensional NPs. The unique inter-NP electronic, photonic, and energy transfer properties of one-dimensional NP assemblies [14,15] are essential not only for the production of novel devices but also for the understanding of fundamental phenomena at the nanometer-scale as well as processes in living organisms [16,17].

Anisotropic assembly of NPs has been achieved with selective modification methods [18], liquid–liquid or gas–liquid interface systems [19], and templating systems [20,21]. Zhang et al. [9] produced one-dimensional assemblies of citrate-coated gold NPs (cAuNPs) by controlling electrostatic interactions and repulsion [22]. Partial exchange of protecting agents on cAuNP surfaces increased the dipolar interaction potential, which induced anisotropic interactions. Anisotropic cAuNP self-assembly on sphere-shaped vesicles of phospholipid bilayers (liposomes) was recently reported [23]. cAuNPs adsorbed on liposome surfaces were initially fixed and did not self-assemble below the phospholipid phase transition temperature (*T*_m_). However, anisotropic cAuNP self-assembly did occur when the phospholipids became fluid above *T*_m_. The decomposition of the citrate layer on the AuNP surface might have induced dipolar interactions between AuNPs, and thus enabled anisotropic assembly. However, the control of anisotropic NP assembly (e.g., control of the number of assembled NPs) has not been sufficiently examined.

Here, the growth of anisotropic cAuNP assemblies by liposome fusion is reported. The relationship between assembly growth and liposome fusion was investigated with time-dependent ultraviolet–visible (UV–vis) absorption, transmission electronic microscopy (TEM), and cryogenic TEM (cryo-TEM). The effect of cAuNP size on liposome fusion was also investigated. Finally, control of the number of cAuNPs in each assembly via regulation of liposome fusion was demonstrated.

## 2. Results and Discussion

### 2.1. Growth of Anisotropic cAuNP_14_ Assemblies on Liposome Surfaces

cAuNPs with average diameters of 14 nm (cAuNP_14_, as determined by TEM (JEOL Ltd., Tokyo, Japan) were prepared by a modified method reported by Frens [24,25]. Liposomes composed of 1,2-dipalmitoyl-*sn*-glycero-3-phosphocholine (DPPC, *T*_m_ = 41.5 °C) were prepared by extrusion as reported previously [23]. The DPPC liposomes and the cAuNP_14_s were mixed at the ratio [cAuNP]/[liposome] = 1.67 at 25 °C in water to induce cAuNP_14_ adsorption on the liposomes. cAuNP_14_ adsorption and anisotropic assembly were confirmed by UV–vis absorption spectroscopy and cryo-TEM, as previously reported [23].

**Figure 1:**
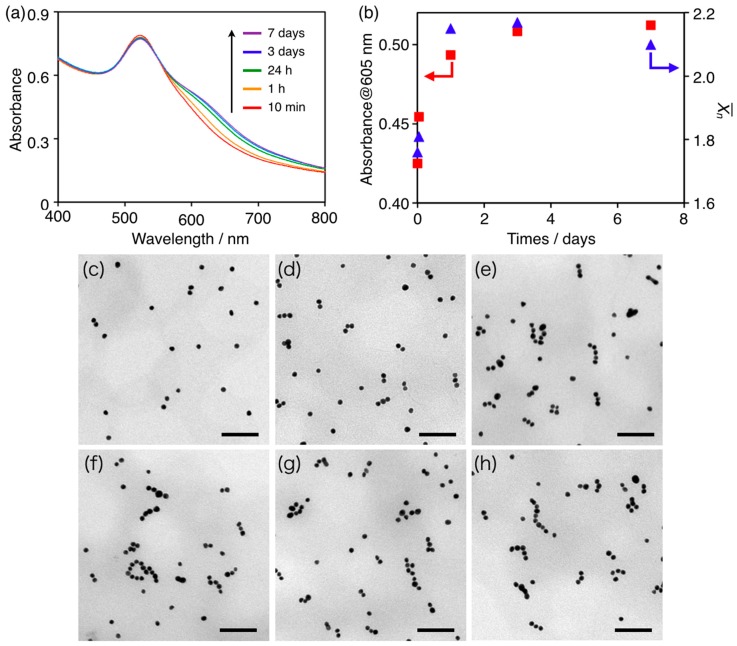
(**a**) Time-dependent UV–vis absorption spectra of cAuNP_14_–DPPC solutions ([cAuNP]/[liposome] = 1.67) heated at 50 °C for 10 min to 7 days; (**b**) Plots of the absorbance at 605 nm (red squares) and the average number of cAuNPs in each assembly ($\bar{X}$_n_, blue triangles) vs. heating time at 50 °C. TEM images of cAuNP_14_–DPPC; (**c**) before heating and after; (**d**) 10 min; (**e**) 1 h; (**f**) 24 h; (**g**) 3 days; and (**h**) 7 days heating at 50 °C. The scale bars are 100 nm.

The time-dependent UV–vis absorption spectrum (SHIMADZU CORPORATION, Kyoto, Japan) of the cAuNP_14_–DPPC composite solution at 50 °C is plotted in Figure 1a. The intensity of the 516 nm plasmon band slightly decreased and a new absorption peak at 605 nm increased over time. The two peaks indicated formation of an anisotropic cAuNP_14_ assembly [23]. The 605 nm absorbance, which was characteristic for anisotropic assembly of cAuNPs [22,23,26,27,28], increased with heating time and became almost saturated after 3 days (red squares in Figure 1b). Furthermore, the 516 nm peak did not shift after 7 days at 50 °C. This indicated that the cAuNP_14_ assemblies maintained anisotropic structures; a red-shift would have indicated an isotropic assembly. When the cAuNP_14_–DPPC composite solution was incubated at 25 °C for 7 days, the 516 nm plasmon band red-shifted slightly and broadened, and there was no peak at 605 nm (Figure S1). These results indicated that anisotropic cAuNP_14_ assemblies gradually grew on the DPPC liposomes by long-term heating above *T*_m_. To confirm the cAuNP_14_ assemblies, TEM images of the cAuNP_14_–DPPC composites were recorded before heating and after 10 min, 1, 24 h, 3, and 7 days heating at 50 °C (Figure 1c–h, respectively). Before heating, the average number of cAuNPs in each assembly ($\bar{X}$_n_) was 1.12, which indicated that individual cAuNPs were isolated on the DPPC liposomes (Figure 1c). After 10 min of heating at 50 °C, anisotropic cAuNP assemblies were observed in the TEM images (Figure 1d) and $\bar{X}$_n_ = 1.76 for each assembly (Figure 1b). When this sample was incubated at 25 °C for 1 week, $\bar{X}$_n_ = 1.72, indicating that cAuNP assemblies were stable and did not grow below *T*_m_. The number of cAuNPs per liposome in the mixed solution was 1.67, indicating that almost all of the cAuNPs on each liposome assembled within 10 min. However, $\bar{X}$_n_ increased to 1.81 and 2.15 after heating for 1 h and 24 h, respectively, and saturated in 3 days (Figure 1b). Hence, almost 1.3 times as many cAuNPs assembled during heating for more than 24 h. This suggested that cAuNP_14_ first assembled on intra-liposomal surfaces and then on inter-liposomal surfaces.

### 2.2. Liposome Fusion during Growth of cAuNP_14_ Assemblies

**Figure 2:**
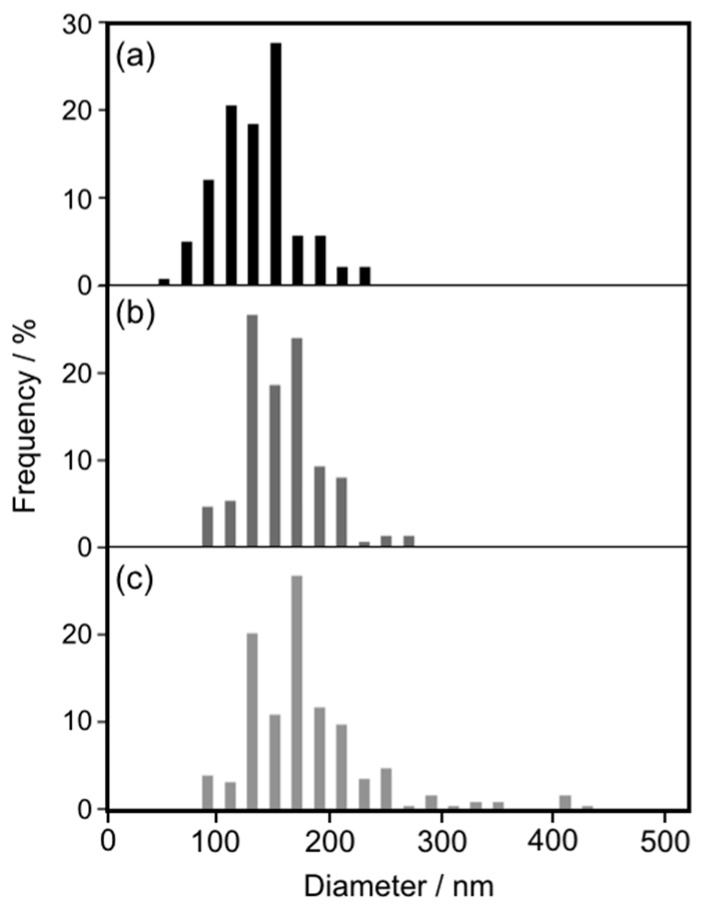
Liposome diameters determined from cryo-TEM images (Figure S2). cAuNP_14_–DPPC (**a**) before and after (**b**) 1 day and (**c**) 7 days heating at 50 °C.

To investigate the growth mechanism of cAuNP_14_ assemblies on intra-liposomal surfaces, DPPC liposome sizes after heating at 50 °C were determined from cryo-TEM images (Figure S2). Histograms of DPPC liposome diameters before and after 24 h and 7 days of heating at 50 °C are shown in Figure 2. The histogram after 24 h (Figure 2b) revealed larger liposomes than those before heating (Figure 2a), which indicated liposome fusion. Fusion was more evident after 7 days of heating, because there were much larger liposomes with average diameters of 165 nm (Figure 2c). The surface areas of liposomes with average diameters of 142 nm (before heating) and 165 nm (after 7 days of heating) were 6.3 × 10^4^ and 8.5 × 10^4^ nm^2^, respectively. This means that an average of 1.35 liposomes fused during 7 days of heating at 50 °C. As mentioned in Section 2.1, almost 1.3 times as many cAuNPs linearly assembled during heating for 24 h or more. This agreement indicated that liposome fusion induced the growth of anisotropic cAuNP assemblies on the membrane surfaces.

### 2.3. Assembly of cAuNPs with Average Diameters of 31 nm on Liposome Surfaces

cAuNPs with average diameters of 31 nm (cAuNP_31_, determined by TEM) were prepared by the stepwise growth method reported by Bastus et al. [29]. As above, DPPC liposomes and cAuNP_31_ were mixed at the ratio [cAuNP]/[liposome] = 1.67 at 25 °C in water to induce cAuNP_31_ adsorption on the liposomes. The adsorption and assembly of cAuNP_31_ on the DPPC liposomes was then investigated.

**Figure 3:**
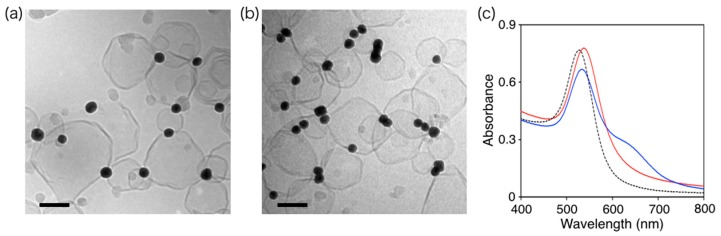
Cryo-TEM images of cAuNP_31_–DPPC ([cAuNP]/[liposome] = 1.67) heated at (**a**) 25 °C and (**b**) 50 °C; The scale bars are 100 nm; (**c**) UV–vis absorption spectra of solutions of cAuNP_31_ (black dashed line), cAuNP_31_–DPPC at 25 °C (red solid line), and cAuNP_31_–DPPC at 50 °C (blue solid line).

Cryo-TEM imaging revealed that the DPPC liposomes were decorated with cAuNP_31_ (Figure 3a), and the number of adsorbed cAuNP_31_ per liposome was determined. The cryo-TEM images also revealed that isolated cAuNP_31_s did not self-assemble on the liposome surfaces below *T*_m_. This was strongly supported by UV–vis spectra of the cAuNP_31_–DPPC solution, where the 530 nm peak attributed to cAuNP plasmon resonance was red-shifted by 10 nm (Figure 3c). The red shift indicated a change in the environment surrounding the cAuNPs [23,30,31,32]. When the cAuNP_31_–DPPC solution was heated above *T*_m_ (41.5 °C), the intensity of the 530 nm plasmon band decreased and a new band appeared at 635 nm in the UV–vis spectrum (Figure 3c). This change was attributed to electric dipole–dipole interactions and plasmon coupling between neighboring gold NPs in the assemblies [33,34,35]. Furthermore, the two peaks indicated formation of an anisotropic cAuNP_31_ assembly that was confirmed by cryo-TEM, shown in Figure 3b. These results were very similar to those for cAuNP_14_. Thus, the cAuNPs assembled on the DPPC liposome surfaces regardless of their average diameter.

### 2.4. Growth of cAuNP_31_ Assemblies by Liposome Fusion

**Figure 4:**
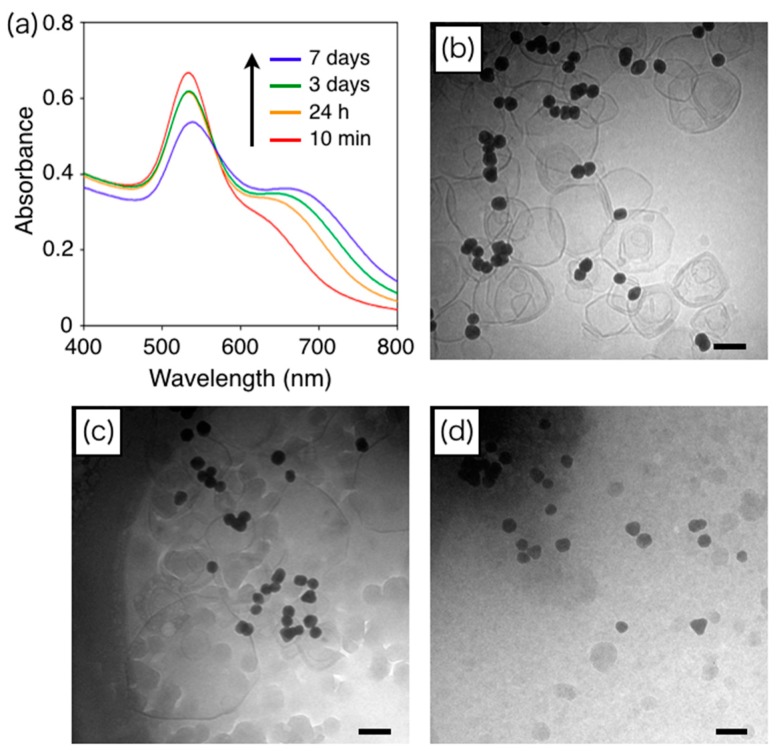
(**a**) Time-dependent UV–vis absorption spectra of cAuNP_31_–DPPC solutions ([cAuNP]/[liposome] = 1.67) heated at 50 °C for 10 min (red line), 24 h (orange line), 3 days (green line), and 7 days (blue line); Cryo-TEM images of cAuNP_31_–DPPC heated at 50 °C for (**b**) 10 min; (**c**) 24 h; and (**d**) 7 days. The scale bars are 100 nm.

A cAuNP_31_–DPPC composite solution was heated at 50 °C to induce the growth of cAuNP_31_ assemblies. Time-dependent UV–vis spectra are shown in Figure 4. The absorbance at 650 nm, which was characteristic of anisotropic cAuNP assembly, increased with heating time. Therefore, anisotropic cAuNP_31_ assemblies also grew on liposome surfaces. For cAuNP_31_–DPPC heated for 7 days (blue line in Figure 4), the absorbance at 400–500 nm was lower than that for shorter heating times, indicating liposome decomposition. The structure of the liposomes during the growth of the cAuNP_31_ assemblies was observed with cryo-TEM imaging for cAuNP_31_–DPPC that was heated for 10 min, 24 h, and 7 days (Figure 4b–d). After 10 min, there were anisotropic cAuNP_31_ assemblies and almost no change in liposome diameters (Figure 4b). After 24 h of heating, the average liposome diameter increased from 121 nm (before heating) to 174 nm (Figure 4c). No liposomes were observed for cAuNP_31_–DPPC that had been heated for 7 days (Figure 4d). Typically, 100 and 200 nm liposomes were the most stable, while those with diameters above 400–500 nm were extremely unstable, especially above *T*_m_ [36]. This indicates that after 7 days of heating, the liposomes in cAuNP_31_–DPPC may become too large to maintain their vesicle structure.

### 2.5. Effect of cAuNP Sizes on Liposome Fusion

**Figure 5:**
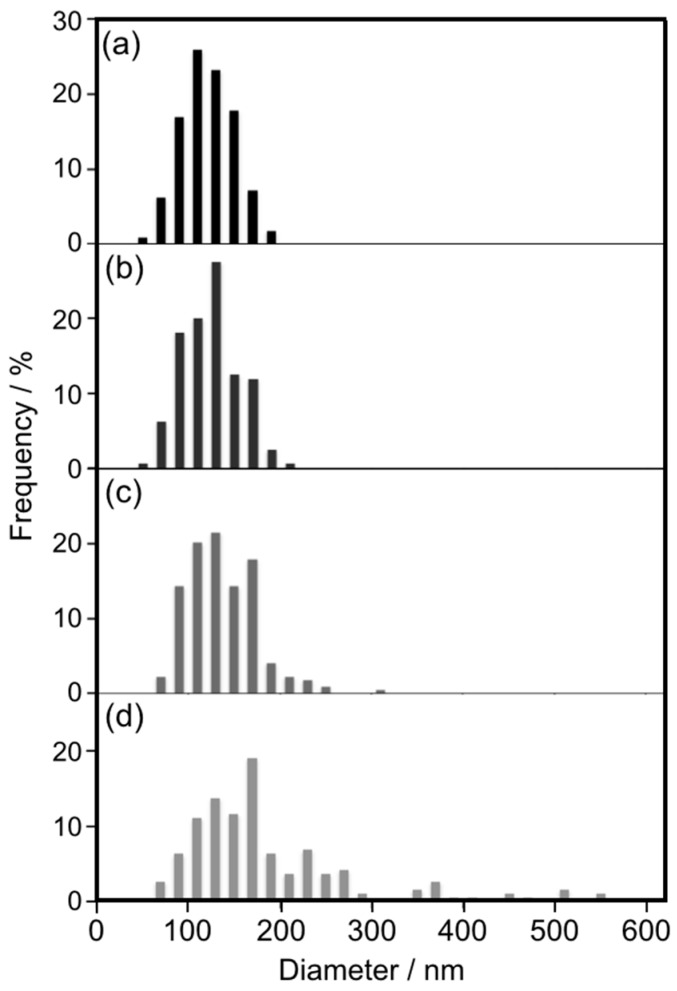
Liposome diameters determined from the cryo-TEM images (Figure S3). DPPC liposomes heated at (**a**) 25 and (**b**) 50 °C for 24 h; (**c**) cAuNP_14_–DPPC and (**d**) cAuNP_31_–DPPC heated at 50 °C for 24 h.

To clarify the effects of cAuNP sizes on liposome fusion, cryo-TEM of DPPC liposomes, DPPC–cAuNP_14_, and DPPC–cAuNP_31_ was performed after 24 h of heating at 50 °C. Histograms of the DPPC liposome diameters are shown in Figure 5. For DPPC liposomes, there was almost no change in average diameter (Figure S3a and Figure 5b). Thus, in the absence of cAuNPs, DPPC liposomes did not fuse after 24 h of heating at 50 °C. In the presence of cAuNP_14_, there was almost no change in the shape of the histogram, but the average diameter of the DPPC liposomes was 8 nm larger than that of the bare DPPC liposomes (Figure S3b and Figure 5c). For the DPPC–cAuNP_31_ composite, there were relatively large liposomes with a 178 nm average diameter (Figure S3c and Figure 5d). The same number of gold nanoparticles were adsorbed on each liposome in the AuNP_14_- and AuNP_31_-liposome composites. Hence, the surface concentration of gold nanoparticles on each AuNP_31_-liposome was larger than that on the AuNP_14_-liposomes, which might have been the reason why AuNP_31_ accelerated liposome fusion relative to AuNP_14_–liposome fusion. This has also been observed for other glue materials in which aggregation and fusion of liposomes was accelerated with increasing size of the glue material [37,38,39]. Therefore, larger cAuNPs accelerate liposome fusion and, accordingly, induce liposome decomposition, as observed for DPPC–cAuNP_31_ after 7 days of heating at 50 °C (Figure 4d).

### 2.6. Control of AuNP $\bar{X}$_n_ in Anisotropic Assemblies by Regulating Liposome Fusion

**Figure 6:**
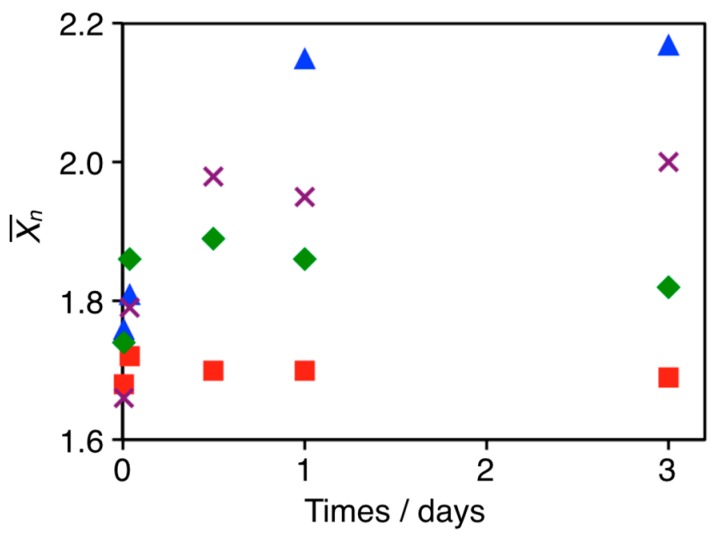
Average number of cAuNPs in each assembly ($\bar{X}$_n_) vs time. cAuNP14–DPPC composite solutions were cooled in an ice bath after heating at 50 °C for 10 min (red squares), 1 h (blue rhombuses), and 12 h (purple crosses). Blue triangles are $\bar{X}$_n_ for cAuNP_14_–DPPC composites heated for 3 days without cooling.

The growth of cAuNP anisotropic assemblies via liposome fusion was demonstrated above. The fusion involved the crosslinking of liposomes by cAuNPs and heating the lipid membrane above *T*_m_. This implied that liposome fusion and cAnNP assembly growth could be regulated by halting solution heating at specific times. To confirm this hypothesis, a cAuNP_14_–DPPC composite ([cAuNP]/[liposome] = 1.67) solution was cooled in an ice bath after 10 min, 1 h, and 12 h of heating at 50 °C. $\bar{X}$_n_ for cAuNP_14_ in each assembly vs incubation time are shown in Figure 6. When the cAuNP_14_–DPPC composite solution was heated at 50 °C for 10 min, $\bar{X}$_n_ increased from 1.12 to 1.68, which was in good agreement with experiments in Figure 1. After cooling in the ice bath, the increase in $\bar{X}$_n_ was stopped and remained 1.70 for 3 days. As already discussed in Figure 1, $\bar{X}$_n_ = 2.17 when the solution was heated at 50 °C for 3 days without cooling. When the composite solution was heated for 1 h at 50 °C, $\bar{X}$_n_ = 1.86 and remained constant for 3 days after cooling in the ice bath. Furthermore, cAuNP_14_ assembly had $\bar{X}$_n_ = 1.98 after being heated at 50 °C for 12 h and remained constant. Thus, the number of cAuNPs in each anisotropic assembly could be precisely controlled by regulating liposome fusion.

## 3. Experimental Section

### 3.1. Materials

1,2-Dipalmitoyl-*sn*-glycero-phosphatidylcholine (DPPC) was purchased from NOF Corp. (Tokyo, Japan). Hydrogen tetrachloroaurate(III)tetrahydrate (HAuCl_4_·4H_2_O) and citric acid were purchased from Kishida Chemical Co., Ltd. (Osaka, Japan) and Wako Pure Chemical Industries, Ltd. (Tokyo, Japan), respectively. All reagents were used as received.

### 3.2. Preparation of cAuNPs with an Average Diameter of 14 nm (cAuNP_14_)

cAuNP_14_ was prepared according to a slightly modified Fens method. In a 300 mL round-bottom flask equipped with a condenser, a 1 mM HAuCl_4_ solution (100 mL) was brought to a rolling boil with vigorous stirring. Rapid addition of 38.8 mM sodium citrate solution (10 mL) to the vortex of the HAuCl_4_ solution resulted in a color change from pale yellow to deep red. Boiling was continued for 10 min, followed by removal of the heating bath and stirring for an additional 15 min. Once the solution reached room temperature, it was filtered through a 0.8 µm membrane. The resulting solution of colloidal particles had a 520 nm absorption maximum, and TEM analysis indicated a particle size of 14.1 nm.

### 3.3. Preparation of cAuNPs with an Average Diameter of 31 nm (cAuNP_31_)

Au seeds were synthesized by heating a solution of 2.2 mM sodium citrate in Milli-Q water (150 mL) with a heating mantle in a 250 mL three-necked round-bottomed flask for 15 min under vigorous stirring. A condenser was used to prevent evaporation of the solvent. After boiling had commenced, 1 mL of 25 mM HAuCl_4_ was injected. The color of the solution changed from yellow to bluish gray and then to soft pink in 10 min. Immediately after Au seed synthesis, the reaction was cooled in the same vessel until the temperature was 90 °C and 1 mL of 25 mM HAuCl_4_ solution was injected. After 30 min, the reaction was complete. This process was repeated twice. The sample was then diluted by extracting 55 mL of the sample and adding 53 mL of Milli-Q water and 2 mL of 60 mM sodium citrate. This solution was then used as the seed solution and the process was repeated. These processes were repeated three times to obtain cAuNP_31_.

### 3.4. Preparation of the DPPC Liposomes

DPPC liposomes were prepared by extrusion. A 130 mM solution of the lipids in chloroform was evaporated under a flow of nitrogen gas until dry. The dried lipid film was then hydrated with 1 mL of Milli-Q water, followed by vortexing for 1 min. The suspension was subjected to eight freeze/thaw cycles using liquid nitrogen and a water bath, respectively, and then extruded through a polycarbonate membrane (100 nm pore size) above *T*_m_.

### 3.5. Preparation of cAuNP_14_–DPPC Liposome Composites

The cAuNP_14_ suspension was centrifuged at 12,000 rpm for 30 min to remove excess citrate and concentrate the cAuNP_14_. The concentrated cAuNP_14_ suspension ([Au] = 18.2 mM, 61.5 µL) was then mixed with the liposomes ([DPPC] = 0.5 mM, 4 mL) at 25 °C. The pH of the mixed solution was 6.7. The ratio of AuNP_14_ to DPPC liposomes was calculated from cryo-TEM images.

### 3.6. Preparation of cAuNP_31_–DPPC Liposome Composites

The cAuNP_31_ suspension was centrifuged at 8000 rpm for 30 min to remove excess citrate and concentrate the cAuNP_31_. The concentrated cAuNP_31_ suspension ([Au] = 26.8 mM, 100 µL) was then mixed with the liposomes ([DPPC] = 0.25 mM, 3 mL) at 25 °C. The pH of the mixed solution was 6.7. The ratio of AuNP_31_ to DPPC liposomes was calculated from cryo-TEM images.

### 3.7. Cryo-TEM

Cryo-TEM samples were prepared by a universal fixation and preparation system (Leica EM CPC, Wetzlar, Germany). To prevent water evaporation from the samples, the isolated chamber was humidified to near saturation prior to introduction of the sample. Droplets of the sample (2–3 µL) were placed on a micro-perforated cryo-TEM grid and then absorbed with filter paper. This formed a 10–300-nm-thick liquid film that freely spanned the micropores on the carbon-coated lace-like polymer layer supported by the metal mesh grid. After a minimum holding time of 30 s, the sample grid assembly was rapidly vitrified in liquid ethane at its melting point (−163 to −170 °C). The purpose of the holding time was to relax any possible flow deformation that may have resulted from the blotting process. The vitreous specimen was maintained in liquid nitrogen until it was loaded into a cryogenic sample holder (Gatan 626-DH, Pleasanton, CA, USA). TEM was performed with a JEM-3100 FEF microscope (JEOL Ltd., Tokyo, Japan) at 300 kV. The electron radiation sensitivity of the sample required a minimal dose system. Images were recorded with a Gatan 794 multiscan digital camera and processed with DigitalMicrograph software (version 3.8.1, Gatan Inc., Pleasanton, CA, USA). The optical density gradients in the background, which were normally ramp-shaped, were digitally corrected by a custom subroutine compatible with DigitalMicrograph.

## 4. Conclusions

The growth of cAuNP linear assemblies on DPPC liposome surfaces was investigated. The cAuNPs assembled on intra-liposomal surfaces within 10 min. Further heating induced cAuNP assembly on inter-liposomal surfaces, resulting in one-dimensional assembly of cAuNPs. Inter-liposomal assembly occurs via liposome fusion, which was confirmed by quantitative analysis of cryo-TEM images. Larger cAuNPs accelerate liposome fusion and the linear cAuNP assembly. This enabled precise control of the number of cAuNPs in each assembly by halting solution heating. These results will enable fabrication of functional materials based on NP assembly as well as investigations of cell functions and disease causality.

## Supplementary Materials

The following are available online at www.mdpi.com/1996-1944/10/11/1317/s1, Figure S1: Time-dependent UV–vis absorption spectra of cAuNP_14_–DPPC solution ([cAuNP]/[liposome] = 1.67) heated at 25 °C for 10 min to 7 days, Figure S2: Cryo-TEM images of cAuNP_14_–DPPC (a) before and after (b) 1 day and (c) 7 days of heating at 50 °C. The scale bars are 100 nm, Figure S3: Cryo-TEM images of DPPC liposomes heated at 50 °C for 24 h in the (a) absence and presence of (b) cAuNP_14_ and (c) cAuNP_31_. The scale bars are 100 nm.

## References

[1] Kelly K.L., Coronado E., Zhao L.L., Schatz G.C. (2003). The optical properties of metal nanoparticles: The influence of size, shape, and dielectric environment. J. Phys. Chem. B.

[2] Mock J.J., Barbic M., Smith D.R., Schultz D.A., Schultz S. (2002). Shape effects in plasmon resonance of individual colloidal silver nanoparticles. J. Chem. Phys..

[3] Lee K.-S., El-Sayed M.A. (2006). Gold and silver nanoparticles in sensing and imaging: Sensitivity of plasmon response to size, shape, and metal composition. J. Phys. Chem. B.

[4] Halas N.J. (2005). Playing with plasmons. Tuning the optical resonant properties of metallic nanoshells. MRS Bull..

[5] Ghosh S.K., Nath S., Kundu S., Esumi K., Pal T. (2004). Solvent and ligand effects on the localized surface plasmon resonance (LSPR) of gold colloids. J. Phys. Chem. B.

[6] Underwood S., Mulvaney P. (1994). Effect of the solution pefractive-index on the color of gold collids. Langmuir.

[7] Miller M.M., Lazarides A.A. (2005). Sensitivity of metal nanoparticle surface plasmon resonance to the dielectric environment. J. Phys. Chem. B.

[8] Kotov N.A., Meldrum F.C., Wu C., Fendler J.H. (1994). Monoparticulate layer and Langmuir-Blodgett-type multiparticulate layers of size-quantized cadmium-sulfide clusters—A colloid-chemical approach to construction. J. Phys. Chem..

[9] Murray C.B., Kagan C.R., Bawendi M.G. (2000). Synthesis and characterization of monodisperse nanocrystals and close-packed nanocrystal assemblies. Annu. Rev. Mater. Sci..

[10] Pileni M.P. (2001). Nanocrystal self-assemblies: Fabrication and collective properties. J. Phys. Chem. B.

[11] Collier C.P., Vossmeyer T., Heath J.R. (1998). Nanocrystal superlattices. Annu. Rev. Phys. Chem..

[12] Xi C., Marina P.F., Xia H., Wang D. (2015). Directed self-assembly of gold nanoparticles into plasmonic chains. Soft Matter.

[13] Tang Z.Y., Kotov N.A. (2005). One-dimensional assemblies of nanoparticles: Preparation, properties, and promise. Adv. Mater..

[14] Maier S.A., Brongersma M.L., Kik P.G., Meltzer S., Requicha A.A.G., Atwater H.A. (2001). Plasmonics—A route to nanoscale optical devices. Adv. Mater..

[15] Maier S.A., Kik P.G., Atwater H.A., Meltzer S., Harel E., Koel B.E., Requicha A.A.G. (2003). Local detection of electromagnetic energy transport below the diffraction limit in metal nanoparticle plasmon waveguides. Nat. Mater..

[16] Walker M.M., Dennis T.E., Kirschvink J.L. (2002). The magnetic sense and its use in long-distance navigation by animals. Curr. Opin. Neurobiol..

[17] Alivisatos P. (2004). The use of nanocrystals in biological detection. Nat. Biotechnol..

[18] Nie Z., Fava D., Kumacheva E., Zou S., Walker G.C., Rubinstein M. (2007). Self-assembly of metal-polymer analogues of amphiphilic triblock copolymers. Nat. Mater..

[19] Yang S., Wang C.-F., Chen S. (2011). Interface-directed assembly of one-dimensional ordered architecture from quantum dots guest and polymer host. J. Am. Chem. Soc..

[20] Le J.D., Pinto Y., Seeman N.C., Musier-Forsyth K., Taton T.A., Kiehl R.A. (2004). DNA-templated self-assembly of metallic nanocomponent arrays on a surface. Nano Lett..

[21] Bae A.H., Numata M., Hasegawa T., Li C., Kaneko K., Sakurai K., Shinkai S. (2005). 1D arrangement of an nanoparticles by the helical structure of schizophyllan: A unique encounter of a natural product with inorganic compounds. Angew. Chem. Int. Ed..

[22] Zhang H., Wang D. (2008). Controlling the growth of charged-nanoparticle chains through interparticle electrostatic repulsion. Angew. Chem. Int. Ed..

[23] Sugikawa K., Kadota T., Yasuhara K., Ikeda A. (2016). Anisotropic self-Assembly of citrate-Coated gold nanoparticles on fluidic liposomes. Angew. Chem. Int. Ed..

[24] Grabar K.C., Freeman R.G., Hommer M.B., Natan M.J. (1995). Preparation and characterization of Au collid monolayers. Anal. Chem..

[25] Frens G. (1973). Controlled nucelation for regualtion of particle-size in monodisperse gold suspentions. Nature.

[26] Dujardin E., Hsin L.B., Wang C.R.C., Mann S. (2001). DNA-driven self-assembly of gold nanorods. Chem. Commun..

[27] Zhong Z.Y., Patskovskyy S., Bouvrette P., Luong J.H.T., Gedanken A. (2004). The surface chemistry of Au colloids and their interactions with functional amino acids. J. Phys. Chem. B.

[28] Jiang L., Guan J., Zhao L., Li J., Yang W. (2009). pH-dependent aggregation of citrate-capped Au nanoparticles induced by Cu^2+^ ions: The competition effect of hydroxyl groups with the carboxyl groups. Colloids Surf. A.

[29] Bastus N.G., Comenge J., Puntes V. (2011). Kinetically controlled seeded growth synthesis of citrate-stabilized gold nanoparticles of up to 200 nm: Size focusing versus ostwald ripening. Langmuir.

[30] Dewi M.R., Laufersky G., Nann T. (2014). A highly efficient ligand exchange reaction on gold nanoparticles: Preserving their size, shape and colloidal stability. RSC Adv..

[31] Sugikawa K., Furukawa Y., Sada K. (2011). SERS-active metal-organic frameworks embedding gold nanorods. Chem. Mater..

[32] Yu C., Varghese L., Irudayaraj J. (2007). Surface modification of cetyltrimethylammonium bromide-capped gold nanorods to make molecular probes. Langmuir.

[33] Takeuchi Y., Ida T., Kimura K. (1996). Temperature effect on gold nanodispersion in organic liquids. Surf. Rev. Lett..

[34] Kreibig U., Genzel L. (1985). Optical-absorption of small metallic particles. Surf. Sci..

[35] Liu K., Lukach A., Sugikawa K., Chung S., Vickery J., Therien-Aubin H., Yang B., Rubinstein M., Kumacheva E. (2014). Copolymerization of metal nanoparticles: A route to colloidal plasmonic copolymers. Angew. Chem. Int. Ed..

[36] Anderson M., Omri A. (2004). The effect of different lipid components on the in vitro stability and release kinetics of liposome formulations. Drug Deliv..

[37] Sideratou Z., Foundis J., Tsiourvas D., Nezis I.P., Papadimas G., Paleos C.M. (2002). A novel dendrimeric “glue” for adhesion of phosphatidyl choline-based liposomes. Langmuir.

[38] Tiriveedhi V., Kitchens K.M., Nevels K.J., Ghandehari H., Butko P. (2011). Kinetic analysis of the interaction between poly(amidoamine) dendrimers and model lipid membranes. Biochim. Biophys. Acta.

[39] Wang X., Mart R.J., Webb S.J. (2007). Vesicle aggregation by multivalent ligands: Relating crosslinking ability to surface affinity. Org. Biomol. Chem..

