# Supplementary material for: Growth of Anisotropic Gold Nanoparticle Assemblies via Liposome Fusion
Source: Materials (Basel). 2017 Nov 17;10(11):1317. doi: 10.3390/ma10111317 (PMC5706264; doi:10.3390/ma10111317)
Supplement: Supplementary file 1 [file materials-10-01317-s001.pdf]

Supplementary Materials

Growth of Anisotropic Gold Nanoparticle Assemblies via Liposome Fusion

Kouta Sugikawa*, Tatsuya Kadota, Kotaro Matsuo, Kazuma Yasuhara, and Atsushi Ikeda*

Department of Applied Chemistry, Graduate School of Engineering, Hiroshima University, Higashi-Hiroshima 739-8527, Japan

Graduate School of Materials Science, Nara Institute of Science and Technology, Nara 630-0192, Japan

FIGURES

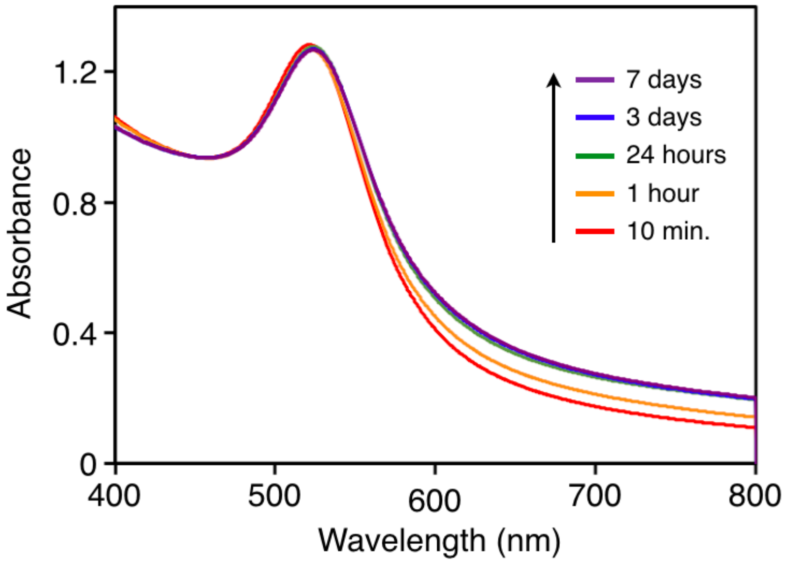

Figure S1. Time-dependent UV–vis absorption spectra of cAuNP14–DPPC solution ([cAuNP]/[liposome] = 1.67) heated at 25 °C for 10 min to 7 days.

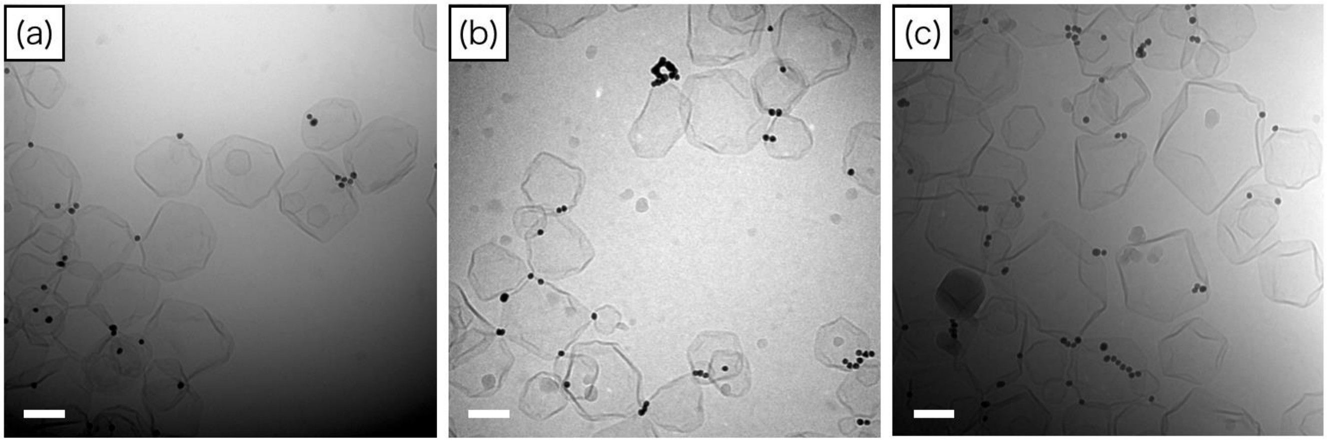

Figure S2. Cryo-TEM images of cAuNP14–DPPC (**a**) before and after (**b**) 1 day and (**c**) 7 days heating at 50 °C. The scale bars are 100 nm.

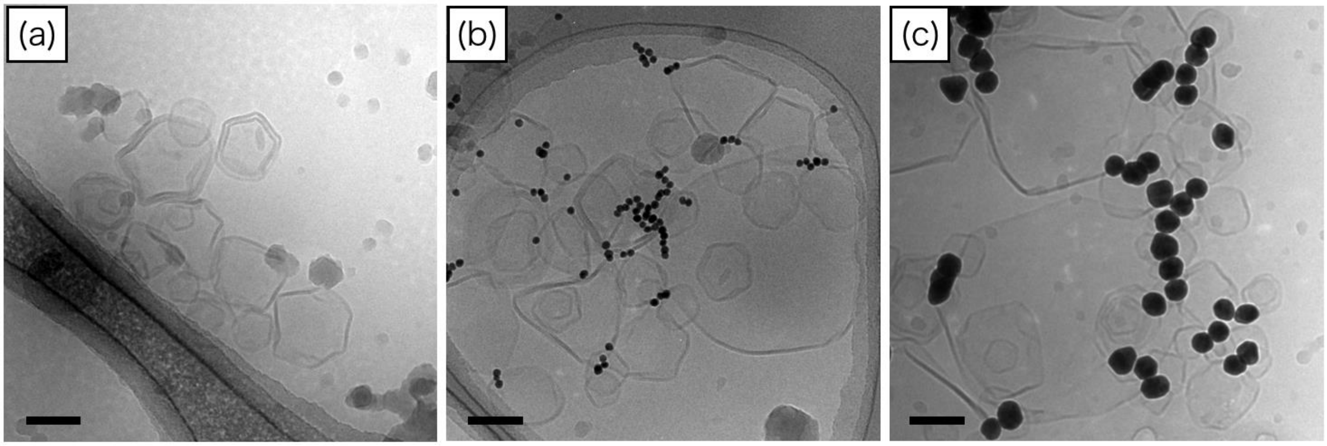

Figure S3. Cryo-TEM images of DPPC liposomes heated at 50 °C for 24 h in the (**a**) absence and presence of (**b**) $cAuNP_{14}$ and (c) $cAuNP_{31}$. The scale bars are 100 nm.
